# Glycan-Induced Transchelation of Gadolinium from Magnetic Resonance Imaging Contrast Agent-Complexes

**DOI:** 10.1021/acs.analchem.4c06624

**Published:** 2025-05-30

**Authors:** Lukasz Polewski, Daria Dymnikova, Weronika Malicka, Maike Lettow, Gert von Helden, Christian Teutloff, Matthias Ballauff, Matthias Taupitz, Robert Bittl, Kevin Pagel

**Affiliations:** † Institute of Chemistry and Biochemistry, Freie Universität Berlin, Altensteinstrasse 23a, 14195 Berlin, Germany; ‡ Department of Molecular Physics Fritz-Haber-Institut der Max-Planck-Gesellschaft, Faradayweg 4-6, 14195 Berlin, Germany; § Institute of Chemistry and Biochemistry, Freie Universität Berlin, Arnimallee 14, 14195 Berlin, Germany; ∥ Department of Radiology, Charité—Universitätsmedizin Berlin, Corporate Member of Freie Universität Berlin and Humboldt-Universität zu Berlin, Charitéplatz 1, 10117 Berlin, Germany

## Abstract

Glycosaminoglycans (GAGs) are linear, highly acidic polysaccharides that serve as essential extracellular matrix components. There has been increasing evidence that GAGs can release gadolinium ions from complexes of magnetic resonance imaging contrast agents. This unintended release of gadolinium might be an initial step leading to gadolinium deposition disease, as observed in some patients after intravenous injection of such contrast agents. However, the molecular details of the release remain poorly understood. In this work, we provide direct evidence for gadolinium binding by GAGs using synthetic model substance Fondaparinux (FPX), a heparin mimetic. We observed FPX–gadolinium complexes in mass spectrometry experiments and electron paramagnetic resonance spectroscopy (EPR) and characterized the binding by EPR, isothermal titration calorimetry, and gas-phase infrared (IR) spectroscopy. Finally, we were able to follow the transchelation process on a molecular level by utilizing collision-induced dissociation experiments.

## Graphical abstract

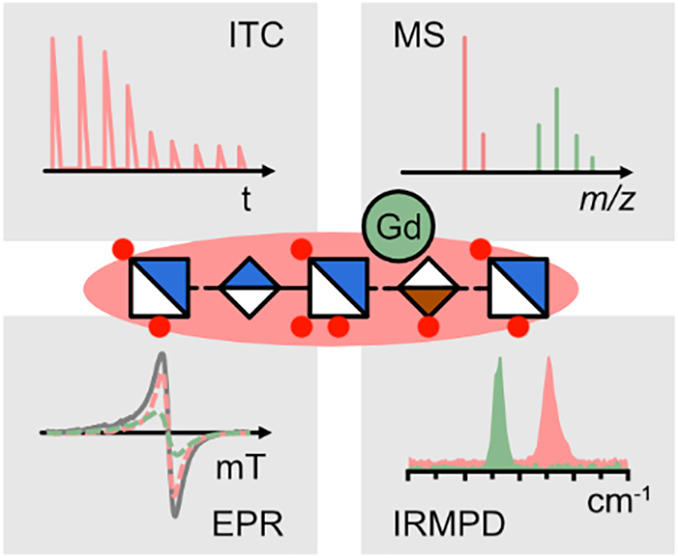

## Introduction

Gadolinium-based contrast agents are frequently used in clinical magnetic resonance imaging (MRI), where after intravenous injection they enhance the recorded signal intensity by reducing the *T*_1_-relaxation time of protons and allow for better detection and delineation of diseased tissue.^1,2^ Due to the high toxicity of free gadolinium ions, as components of the contrast agents, they are bound in complexes using strong chelating, low molecular weight compounds like pentetic acid (DTPA) or dodecane tetraacetic acid (DOTA). However, it is known that, depending on the patient and the used contrast agent, a varying fraction of the injected gadolinium remains in the body.^3–5^ Contrast agents with linear complexes generally show more gadolinium loss than those with macrocyclic complexes.^6–8^ This is attributed to the higher binding affinity of gadolinium to the macrocyclic ligand and the increased kinetic stability.^9^ The most severe outcome of gadolinium retention was nephrogenic systemic fibrosis, a condition observed in patients with severe renal insufficiency and directly linked to gadolinium-based contrast agent injection.^10,11^ Meanwhile, gadolinium retention is also observed in tissues of patients with normal renal function. Some of them develop a variety of symptoms, which is called gadolinium deposition disease.^12^

The chemical nature and the environment of the gadolinium that is retained in the body remain largely unknown.^13,14^ It has been proposed that gadolinium cations are initially released from the contrast agent via transmetalation with a competing metal ion such as Zn^2+^, Cu^2+^, or Fe^3+^.^15–17^ This mechanism has been shown for linear contrast agents; the release from macrocyclic contrast agents, on the other hand, remains poorly understood. Interestingly, MRI measurements revealed a delayed increase of signal intensity in inflamed tissue after injection of contrast agents, which is often referred to as late gadolinium enhancement and which may be caused at least partially by retention of released gadolinium or of the intact contrast agent complex.^18^ A possible explanation is the binding of gadolinium to an unknown macromolecular species. Glycosaminoglycans (GAGs), as a major component of the extracellular matrix, have recently been identified as one of the likely binding partners.^19,20^ GAGs are linear, highly acidic polysaccharides, which are physio- and pharmacologically highly relevant. Their repetitive linear backbone makes them appear structurally simple at first glance, but sulfation isomers and epimerization of hexuronic acid residues lead to an enormous structural diversity.^21^ As polyelectrolytes GAGs are known to bind a variety of metal ions,^22–25^ which can lead to conformational changes in both the monosaccharides^26^ and the higher-order structure of the GAGs.^23,27^ In some cases, the metal ion can also mediate interactions with otherwise unfavorable binding partners.^28–30^

Here we investigate the binding of gadolinium to Fondaparinux (FPX), a heparin-derived oligosaccharide that is clinically used for anticoagulation.^31^ It is a small and easily accessible molecule that structurally resembles the ATIII binding domain of heparin (Figure 1a).^32^ With a total of eight sulfate groups and two carboxylic acids, it exhibits all potential gadolinium binding sites present in heparin and heparan sulfate, which makes it an ideal model substance. The experiments reveal the binding of gadolinium ions to FPX with a μM affinity and their release from the chelating complex in the presence of FPX.

**Figure 1:**
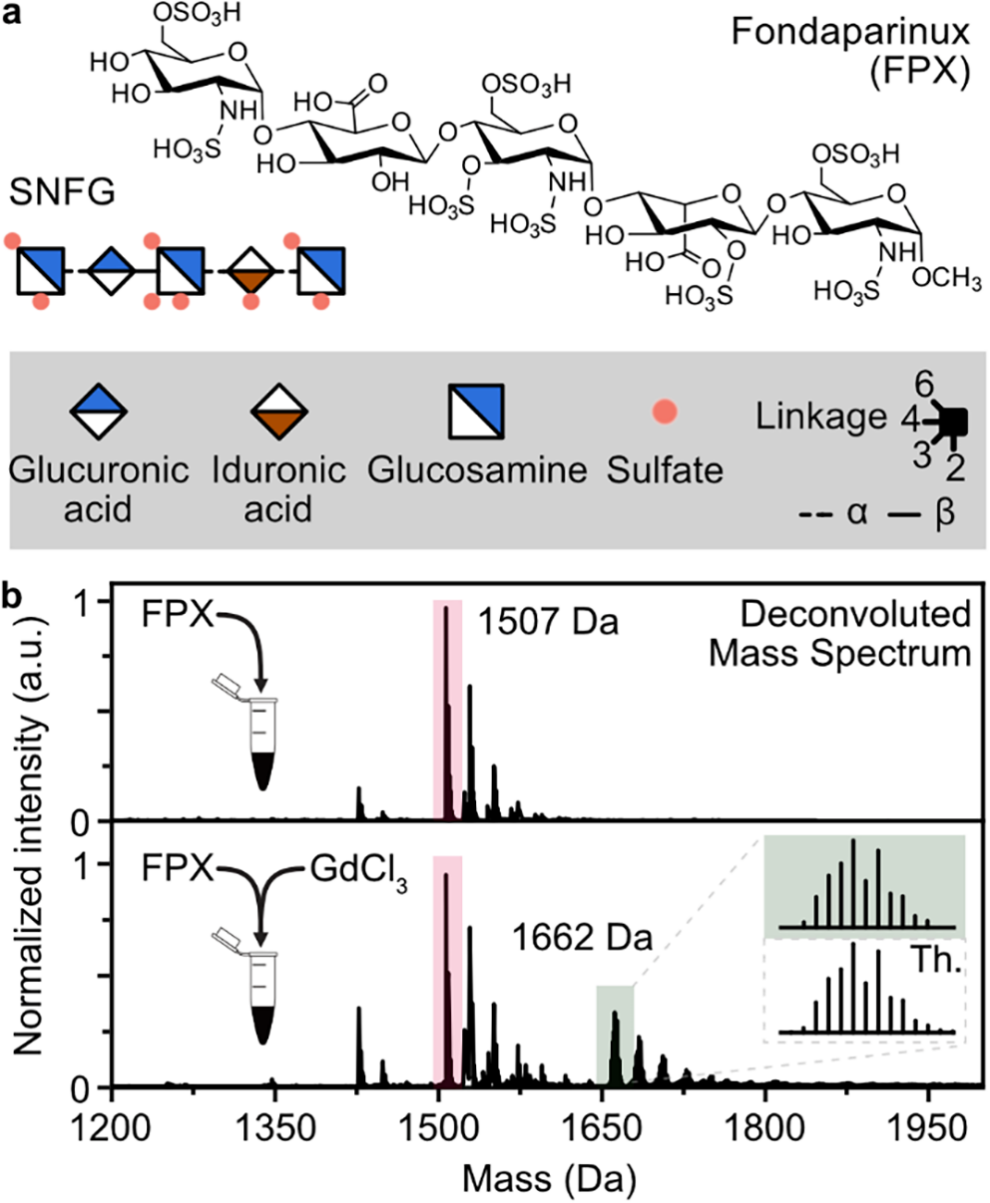
(a) Fondaparinux (FPX), a synthetically derived highly sulfated pentasaccharide, is shown as a chemical structure and depicted using the symbol nomenclature for glycans (SNFG).^37^ (b) Deconvoluted mass spectrum of FPX (highlighted red) and an equimolar FPX–GdCl_3_ mixture. Both mass and the characteristic isotope pattern of gadolinium match the theoretical mass and pattern of the FPX–gadolinium complex (highlighted green).

## Experimental Methods

### Sample Preparation

Commercially available fondaparinux-sodium, solvents, and chemicals were purchased from Sigma-Aldrich (St Louis). For mass spectrometry (MS) applications, fondaparinux-sodium was desalted using a 5 mL Cytiva (Washington, DC) HiTrap desalting column at a 1 mL/min flow rate (water) with a Knauer (Berlin, Germany) FPLC system. For MS analysis, aqueous fondaparinux stock solution (1 mM) was further diluted prior to use with 100 mM ammonium acetate in water to yield 10–50 μM analyte solutions. For the infrared multiple photon dissociation (IRMPD) measurements, fondaparinux was diluted in water/methanol. (1:1; v/v). The isothermal titration calorimetry (ITC) experiments were performed in 20 mM sodium acetate buffer at pH 5.2 and a temperature of 25 °C. The electron paramagnetic resonance spectroscopy (EPR) samples were prepared from the stock solutions using an aqueous solution of 10 mM GdCl_3_, 10 mM FPX with Milli-Q water, and glycerol (for the low temperature measurements). Commercially available gadolinium chloride hexahydrate (GdCl_3_, Sigma-Aldrich) and Fondaparinux sodium salt (FPX, Sigma-Aldrich) were used for stock solutions.

### EPR Spectroscopy

Room temperature continuous wave EPR (cwEPR) measurements at the W-band (94 GHz) were performed on an Elexsys E680 EPR spectrometer using a Teraflex EN600-1021H probe head (both Bruker Biospin, Karlsruhe, Germany). The samples were filled into quartz capillaries (Vitrocom, Mountain Lakes) with 0.25/0.15 mm outer/inner diameters (OD/ID) that were inserted into larger capillaries with 0.8/0.3 mm (OD/ID). All EPR spectra were analyzed with the MATLAB (The MathWorks GmbH, Ismaning, Germany) software using the EasySpin toolbox.^33^ CwEPR spectra were corrected for dispersion/absorption phase and a background (third-degree polynomial).

Cryogenic temperature (10 K) measurements at the X-band (9.7 GHz) were performed on a Bruker ElexSys E680 spectrometer equipped with Oxford CF935 helium cryostats using a Bruker MD5 probe head. The temperature was controlled by an ITC503 (Oxford Instruments, Oxford, U.K.). Quartz capillaries (QSIl, Ilmenau, Germany) with 3.9/3.0 (OD/ID) were filled with a 1:1 water/glycerol sample solution and rapidly frozen in ethanol. Spin echo decays were fitted with stretched exponentials.

### ITC Measurements

Experiments were performed on a MicroCal VP-ITC (Malvern Panalytical GmbH, Germany). Each sample was degassed at the temperature of the respective experiment before the measurement. A total of 290 μL of 2 mM GdCl_3_ was titrated into the 1043 μL sample cell with 0.05 mM polysaccharide solution, with 56 or 46 successive injections in 5 or 6 μL steps, with a stirring rate of 307 rpm and a time interval of 300 s between each injection. For all experiments, the instrument software (MicroCal PEAQ-ITC Analysis) was used for baseline adjustment, peak integration, and normalization of the reaction heats with respect to the molar amount of injected ligand, as well as for data fitting and binding parameter evaluation. The binding experiments were corrected for the heat of GdCl_3_ dilution, which had been determined separately (GdCl_3_ titration into buffer).

### Mobility-Selected IR Spectroscopy

For the IRMPD spectroscopy, the in-house constructed drift-tube ion mobility-mass spectrometer “iMob” was used. Ions are produced by nanoelectrospray ionization from a Pd/Pt-coated borosilicate capillary and are transferred and stored in an entrance funnel. Afterward, the ions are released by 150 μs long pulses into a drift-tube filled with helium buffer gas (∼5 mbar) and travel through the drift-tube under the influence of a weak electric field (10–20 V/cm). After the ion mobility cell, the ions are mass-selected using a quadrupole mass filter, and their arrival time distributions (ATD) can be recorded by measuring the time-dependent ion current of the mass-selected species.

IRMPD spectra are recorded by selecting a drift time window using electrostatic deflection prior to mass selection in the quadrupole. The ion mobility and *m*/*z* selected ion cloud are then further irradiated by a 60–100 mJ 10 μs pulse of IR photons. Photofragmentation is detected in a time-of-flight mass analyzer and the IR spectra are obtained by plotting the fragmentation yield against the wavenumber of the tunable IR laser. The scanning was performed by wavenumber steps of 2 cm^–1^ and 25–75 averages were used per recorded point.

The tunable light in the mid-IR range is supplied by the free-electron laser of the Fritz Haber Institute and transported to the instrument via an evacuated beamline. The last 2 m of the beamline are purged with dry nitrogen to avoid water absorption.

### MS Measurements

MS measurements were performed on a Bruker timsTOF Pro instrument using the MS only mode. Settings were optimized to prevent unwanted fragmentation with a quadrupole ion energy of 2.5 V, collision-induced dissociation (CID) voltage of 7 V, collision gas flow rate of 65%, prepulse storage time of 9 μs, and transfer time of 100 μs. MS-CID measurements were performed on a Waters Synapt G2-S. Deconvolution of MS data was performed with an in-house developed program.

## Results and Discussion

After the addition of a gadolinium salt solution to FPX, an FPX–gadolinium complex can be observed using negative electrospray ionization (ESI)-MS. The complex is visible in multiple charge states between −2 and −5. Deconvolution of the mass spectrum provides a clearer picture (Figure 1b). The FPX–gadolinium species can be readily identified based on the unique isotopic pattern of gadolinium. Given the tendency of the ESI process to induce nonspecific ionic adduct formation, particularly affecting the quantity of FPX bound to gadolinium, solvent data is essential for confirming the observed gadolinium binding.

To determine the fraction of FPX-bound gadolinium ions, we performed W-band continuous wave EPR (cwEPR) experiments. EPR spectra for gadolinium complexes in an aqueous solution at room temperature show single Lorentzian line spectra. In the absence of FPX, a peak-to-peak line width of about 7.4 mT is observed. The addition of FPX to the gadolinium solution reduces the line width of the spectrum compared to free gadolinium ions to about 4.5 mT (Figure 2a). The decrease in line width is gradual until a molar ratio of about 1:2 gadolinium/FPX and remains constant with further increasing FPX concentration. A closer inspection of the spectral line shapes indicates the presence of two Lorentzian components. One possible origin for the line width change might be the viscosity change upon FPX addition. However, a 4-fold dilution of the 1:1 gadolinium/FPX solution does not alter the line width, albeit the viscosity is again the same as that of the 1:0.25 gadolinium/FPX sample (Figure 2b) and a viscosity effect can be excluded. Previously, variations in the Lorentzian line width have been observed for gadolinium in different environments. These changes were attributed to the altered modulation of the zero-field splitting (ZFS) and the resulting different *T*_2_ relaxation times in liquid solution.^34–36^

**Figure 2:**
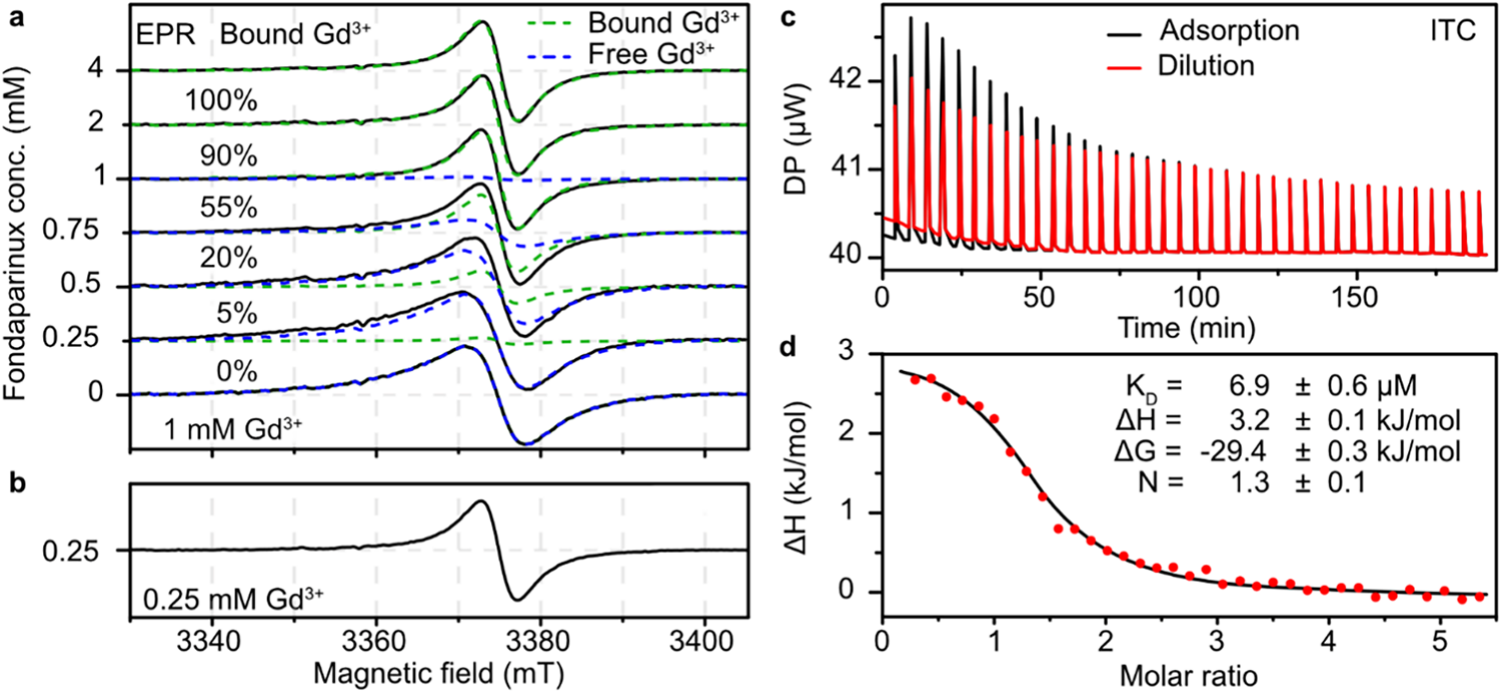
(a) W-band (94 GHz) room temperature cwEPR measurements on 1 mM Gd^3+^ binding to FPX in water. The decomposition of the spectra (black) to free Gd^3+^ (aqua complex, blue) and Gd^3+^ bound to FPX (green) spectra are shown. The obtained fractions of free and bound Gd^3+^ are also shown (rounded to 5%). (b) W-band EPR spectrum of the 4× diluted 1:1 Gd/FPX solution. (c) Endothermic raw data of the gadolinium–FPX ITC experiments. Both adsorption (black) and dilution (red) are shown. (d) Integrated amount of heat released from the titration plotted against the molar ratio of the components (Gd^3+^/FPX).

Taken together, these findings suggest that the weights of the two spectral components correspond to the relative amounts of aqueous gadolinium and gadolinium bound to FPX, respectively. The fitted weights of the components are given in Figure 2a, and fits are represented as dashed lines, individually. A fit of the FPX-bound gadolinium fraction against the gadolinium/FPX ratio by a logistic function yields a concentration ratio of about *N* = 1.5 ± 0.2 for the equilibrium between FPX-bound and unbound gadolinium (Figure S1). From the EPR observed fraction of FPX-bound gadolinium, we can estimate the number of gadolinium ions per FPX molecule. At a 1:1 gadolinium/FPX ratio, 90% of the gadolinium ions are bound per FPX. Loading of FPX with more than one gadolinium ion would result in the broadening of the cwEPR lines due to gadolinium–gadolinium interactions. However, the large observed line width prevents an interpretation of the cwEPR in terms of gadolinium–gadolinium distance. This problem can be overcome by the use of pulsed EPR at low temperatures. The phase memory time of gadolinium (*T*_M_) in a two-pulse Hahn echo sequence is strongly concentration-dependent for 1 and 10 mM gadolinium solutions (Figure S2)^38^ or the 10 mM gadolinium solution the *T*_M_ is drastically shortened due to the roughly halved average distance (*r*_Gd–Gd_ ∼6.6 nm for 1 mM and *r*_Gd–Gd_ ∼3 nm for 10 mM solution) and a concomitant 10-fold increase of the dipolar interaction.

The addition of FPX leads to a noticeable decrease in *T*_M_ compared with the 1 mM solution but substantially longer compared to the 10 mM solution. The signal decay is virtually indistinguishable for FPX concentrations of 1 and 4 mM. This in turn argues against a substantial contribution of FPX loaded with two or more gadolinium ions, since they would be at distances smaller than about 3 nm (approximate length of FPX),^39^ which should lead to an even shorter *T*_M_ than for the 10 mM gadolinium solution.

Isothermal titration calorimetry (ITC) was performed on the FPX–gadolinium model system to access additional thermodynamic parameters. Figure 2c,d depict the ITC thermogram and the respective fit for heat injection amounts plotted against the molar ratios of the two reactants. From this data, the binding energy and affinity of the complex can be determined. A pronounced interaction between gadolinium ions and FPX is evident, with a binding energy (Δ*G*) of −29.4 kJ/mol and a significant binding affinity with a *K*_D_ measured at 6.9 μM. Despite an unfavorable enthalpy change (Δ*H* = 3.2 kJ/mol) associated with the binding of gadolinium ions to FPX, this thermodynamic imbalance is counteracted by a robust entropic contribution (*T*Δ*S* = 32.6 kJ/mol). The stoichiometry of the binding complex *N* = 1.3 ± 0.1, suggests the presence of multiple binding sites for gadolinium ions on an FPX molecule, which agrees well with the *N* = [Gd^3+^]/[FPX] = 1.5 ± 0.2 equilibrium stoichiometry found in the EPR measurements. The binding affinity of the gadolinium–FPX complex, with a dissociation constant of 6.9 μM, is low compared to most contrast agent complexes, which exhibit affinities more than 10 orders of magnitude higher. Longer GAG chains are highly multivalent and are therefore expected to exhibit a considerably higher binding affinity toward gadolinium than FPX.^40^ However, the binding affinity alone is likely not the major driving force for gadolinium retention in patients who have received a gadolinium-based contrast agent. Instead, it is expected to be a combination of the affinity and the high local concentration of GAGs in the involved tissues.

To investigate the binding of gadolinium to FPX on a molecular level, ion mobility spectrometry (IMS) coupled to infrared multiple photon absorption dissociation (IRMPD) spectroscopy was performed. Both, IRMPD^41,42^ and IMS,^43^ have been successfully applied to probe noncovalent interactions of biomolecules and their structures in the gas phase. The binding of gadolinium to FPX is likely mediated through the eight possible negatively charged sulfates and two uronic acids. We analyzed the [FPX + Gd]^4–^ complex and a [FPX + 3Na]^4–^ complex as a control.

The IMS arrival time distribution (ATD) of the gadolinium complex shows two conformational features (Figure 3a), with the compact conformer as the major component. The triple sodium complex shows a qualitatively similar ATD but instead has the extended rather than the compact conformer as the major component. This difference is likely due to the chelation of gadolinium by FPX, which stabilizes the compact conformers. In sodium adducts, on the other hand, the cations are evenly distributed along the saccharide chain, leading to an overall more extended arrangement.

**Figure 3:**
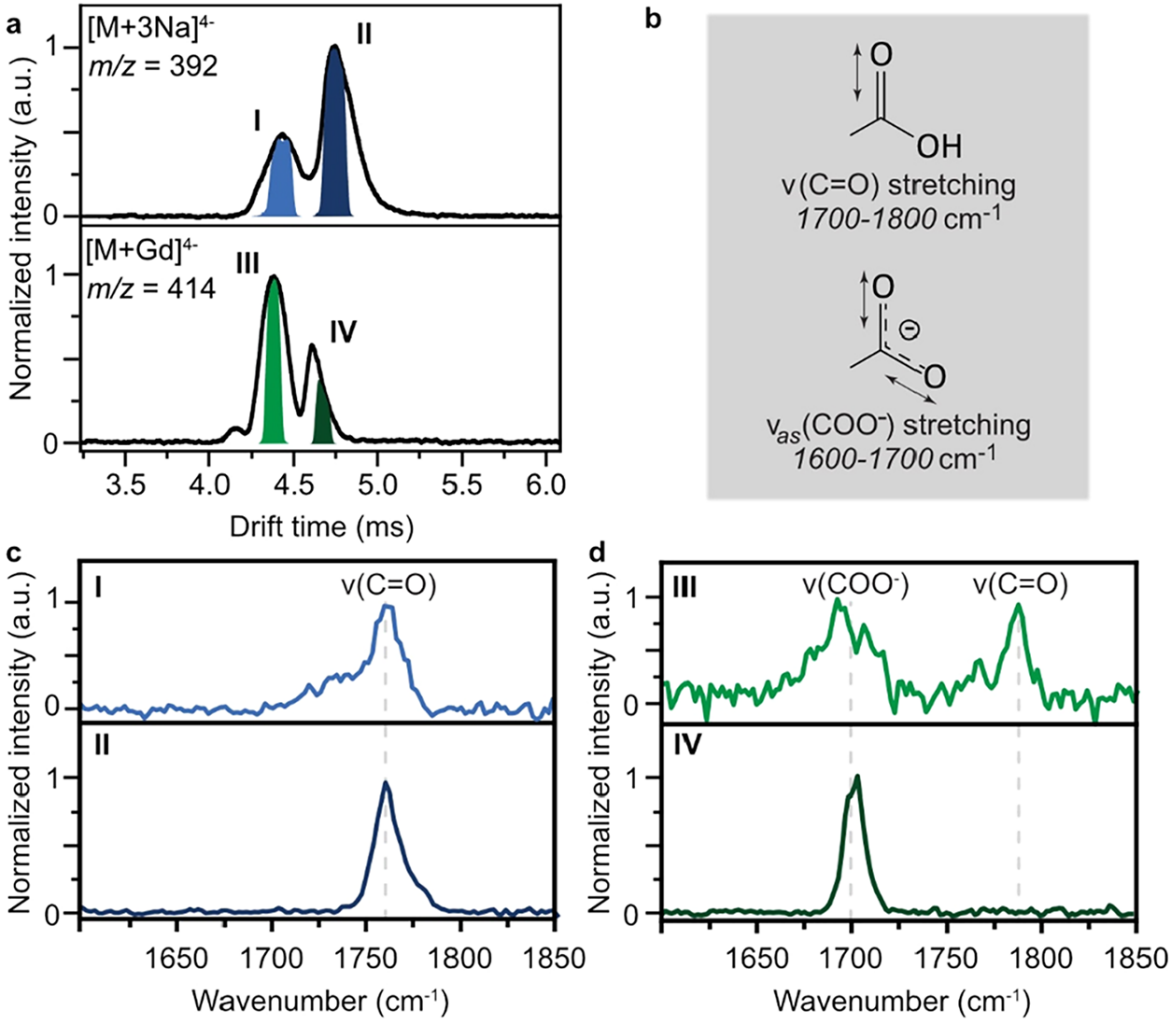
IMS-IRMPD experiments of the FPX–cation complexes. (a) ATD of the triple sodium (top) and the gadolinium (bottom) complex of FPX. The highlighted areas were isolated to perform mobility and mass-selected IRMPD spectroscopy. (b) Characteristic stretching vibrations of carboxylic acids and carboxylates, commonly visible between 1700 and 1800 and 1600–1700 cm^–1^, respectively. (c) IRMPD spectra of the triple sodium adduct. Both isolated conformational groups, I and II, show a clear signal corresponding to the carboxylic acid stretching vibration, indicating no direct participation of the carboxylic acid in FPX-binding. (d) IRMPD spectra of the FPX–gadolinium complex. The compact conformer group III showed two absorption bands corresponding to an asymmetric carboxylate stretching vibration at 1715 cm^–1^ and a carboxylic acid stretching vibration at 1790 cm^–1^. The extended conformer IV showed only a stretching vibration corresponding to the carboxylate, indicating the binding participation of both carboxylic acids.

The compact and extended conformers of the sodium and gadolinium complexes were isolated as highlighted in Figure 4b and IRMPD spectroscopy between 1600 and 1850 cm^–1^ was performed on the selected species. The vibrations of the carboxylic acid and its conjugated base, the carboxylate, from the two uronic acids of FPX are easily distinguishable (Figure 3b). Carboxylic acids can be readily identified by their stretching vibration (v­(C=O)), which most commonly appears between 1700 and 1800 cm^–1^.

**Figure 4:**
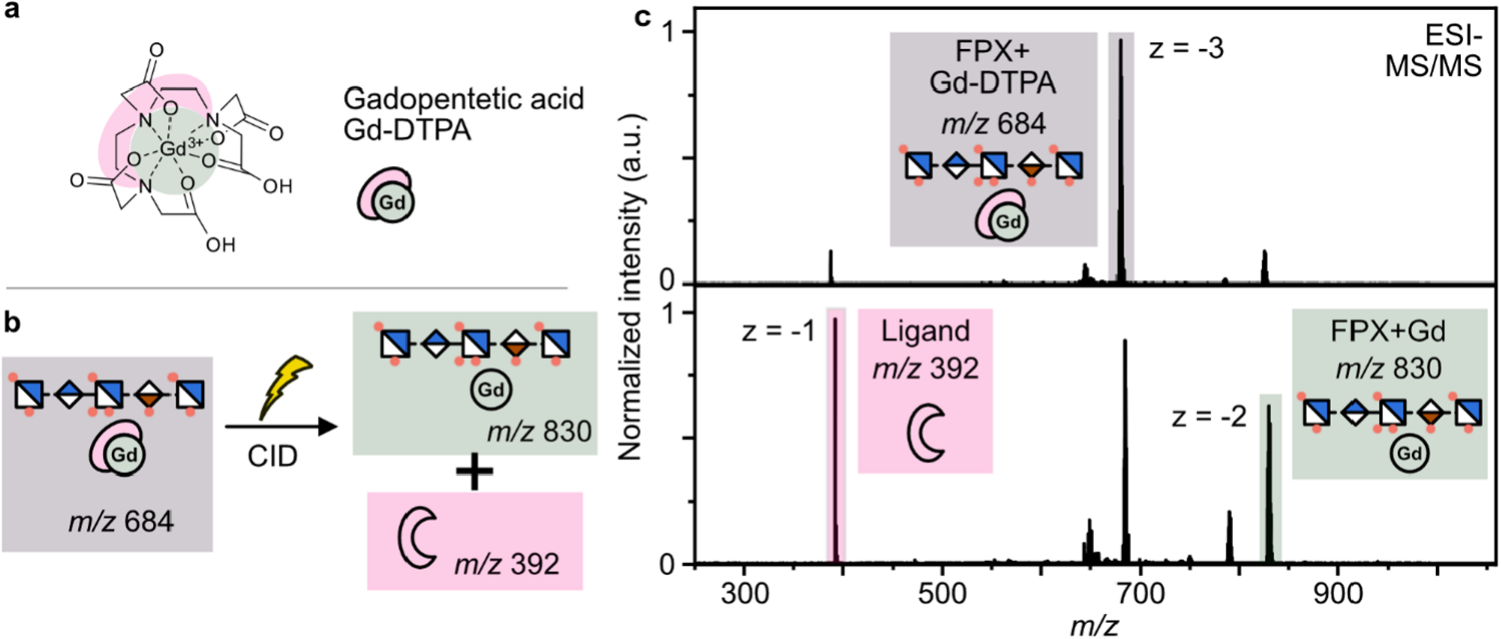
(a) Gadopentetic acid, a linear gadolinium-containing complex, is the active pharmaceutical ingredient of the contrast agent Magnevist. (b) Schematic fragmentation of the FPX–gadopentetic acid complex to the free ligand and the FPX–gadolinium complex. (c) Isolated mass spectrum of the FPX–gadopentetic acid complex (highlighted gray) and its CID-MS/MS-spectrum. The free ligand is highlighted in red, and the FPX–gadolinium complex is in green.

In contrast, the carboxylate is characterized by an asymmetric stretching vibration (v_as_(COO^–^)) between 1600 and 1700 cm^–1^.^44–46^ Previous reports showed that charges on GAGs preferably reside at the sulfate groups, likely due to their higher acidity.^46,47^ Therefore, the presence of a carboxylate might indicate participation of the group in the binding of gadolinium.

A charge on the carboxylic acid could also result from an unfavorable charge position on the sulfates due to an increasing Coulomb repulsion from the additionally introduced negative charges. As the charges of the used control sodium complex mimic the number of charges in the gadolinium complex, with the difference that sodium is known to bind nonspecifically, we can probe a possible influence of Coulomb repulsion.

Both the compact and extended conformers of the sodium complex (I and II) yield very similar spectra with an absorption maximum at 1760 cm^–1^ matching that of a carboxylic acid (Figure 3c). This confirms the assumption that the sodium binding site is mainly governed by the charges positioned on the sulfates. For the compact conformer of the gadolinium complex (III), two absorption bands can be observed at 1700 and 1780 cm^–1^, matching stretching frequencies of a carboxylate and a carboxylic acid, respectively (Figure 3d). Previous NMR experiments with calcium on FPX showed a similar behavior, where calcium preferably binds to iduronic acid.^48^ The extended conformer of the gadolinium complex (IV), on the other hand, only shows a single absorption band at 1700 cm^–1^, indicating a binding on both carboxylic acids, once again showing the specificity, with the gadolinium position determining the charge site.

ESI-MS of GAGs in the presence of gadolinium-based contrast agents yields noticeable signals of GAG–contrast agent complexes, which exhibit a very intriguing fragmentation behavior. For gadopentetic acid (Figure 4a), the active pharmaceutical ingredient of Magnevist, and FPX the complex appears at a triply negatively charged ion at *m*/*z* 684. Activating the complex via collision-induced dissociation (CID) results in two major fragments at *m*/*z* 392 and 830 (Figure 4b). Surprisingly, a transchelation in which the gadolinium remains bound to FPX (*m*/*z* 830) is released, while the ligand of Magnevist (pentetic acid, *m*/*z* 392) is released.

Minor fragments can also be observed at *m*/*z* 663 and 790, resulting from neutral loss of sulfate from the intact FPX–contrast agent and the FPX–gadolinium complexes, respectively (Figure 4c). Similar transchelation processes can be observed with the complexes of other contrast agents, such as gadodiamide and the macrocyclic contrast agent gadobutrol, albeit to a lower extent. As an alternate fragmentation path to transchelation, the FPX–contrast agent complexes can undergo dissociation into the intact contrast agent complex and FPX (Figure S3). The observed difference in dissociation behaviors is likely not connected to the contrast agents’ kinetic stability, given that collision-induced dissociation (CID) is a thermal fragmentation method. Before dissociation or transchelation, stable FPX–contrast agent complexes such as the gadodiamide complex tend to lose one or more sulfates, which likely influences the fragmentation behavior. The loss of sulfates has two major effects on the system: (a) the loss of an additional binding partner for the gadolinium ion and (b) the loss of an additional acidic proton. Considering the previous results, which revealed a contribution of carboxylic acids to gadolinium binding and a considerable difference between the binding affinities of the contrast agent and the FPX–gadolinium complex (log *K*_D_ −20 for Magnevist, −5 for FPX–gadolinium), the loss of an acidic proton is likely the main driving force.

Even though it is unclear to what extent these complexes occur in solution, the influence of acidic protons will have a similar effect on the relative stability of contrast agent complexes and GAG–gadolinium complexes. This suggests that the transchelation of gadolinium ions from the contrast agent complex to GAGs may be more favorable in acidic environments, such as those found in inflamed tissues,^49^ where late gadolinium enhancement is known to be more prevalent and even frequently utilized for diagnostic purposes.^50–52^

## Conclusions

It has been known that in MRI contrast agents, gadolinium ions can be released *in vivo* from their chelates; GAGs have been proposed as one of the potential binding partners. We observed gadolinium binding to the heparin-mimetic FPX. With a variety of condensed- and gas-phase methods, we characterized the binding. ITC and EPR measurements showed a low micromolar dissociation constant and a stoichiometry of *N* = ∼1.3 for the formed FPX–gadolinium complex. Therefore, transchelation of gadolinium from the contrast agent complex to GAGs is likely governed by a combination of the rather moderate affinity and a high local concentration of GAGs in the involved tissue. Gas-phase infrared spectroscopy experiments provided further molecular insights and revealed a preferable binding of gadolinium to the carboxylic groups. A high degree of GAG sulfation is therefore not the only driving force for gadolinium binding to GAGs as shown by Werner et al.^53^ Finally, the formation and dissociation of complexes formed by GAGs and intact contrast agent complexes was observed using mass spectrometry. The dissociation of these complexes in the mass spectrometer was found to be largely determined by the availability of acidic protons. A complex with a high number of acidic protons favorably transchelates, leading to the formation of GAG–gadolinium complexes. In combination with the previous results, this provides a first molecular explanation for why the late gadolinium enhancement commonly observed in MRI is more prevalent in acidic tissue. Additionally, we provided a fundamental understanding of gadolinium binding to the ubiquitous GAGs present in, for example, the human body.

## Supplementary Material



## Notes

Additional experimental EPR and MS data on the FPX–Gd complex and the dissociation of GAG–contrast agent complexes (PDF)
