# Supplementary material for: Glycan-Induced Transchelation of Gadolinium from Magnetic Resonance Imaging Contrast Agent-Complexes
Source: Anal Chem. 2025 May 30;97(22):11436–42. doi: 10.1021/acs.analchem.4c06624 (PMC12163887; doi:10.1021/acs.analchem.4c06624)
Supplement: Supplementary file 1 [file ac4c06624_si_001.pdf]

Supporting information “Glycan-Induced Transchelation of Gadolinium from Magnetic Resonance Imaging Contrast Agent-Complexes”

Lukasz Polewski,[a,b] Daria Dymnikova,[c] Weronika Malicka,[a] Maike Lettow,[b] Gert von Helden,[b] Christian Teutloff,[c] Matthias Ballauff,[a] Matthias Taupitz,[d] Robert Bittl*[c] and Kevin Pagel*[a,b]

[a] L. Polewski, W. Malicka, Prof. M. Ballauf, Prof. K. Pagel
Institute of Chemistry and Biochemistry
Freie Universität Berlin
14195 Berlin, Altensteinstrasse 23a, Germany
E-mail: kevin.pagel@fu-berlin.de

[b] L. Polewski, Dr. M. Lettow, Prof. G. v. Helden, Prof. K. Pagel
Department of Molecular Physics
Fritz-Haber-Institut der Max-Planck-Gesellschaft
14195 Berlin, Faradayweg 4-6, Germany

[c] D. Dymnikova, Dr. C. Teutloff, Prof. R. Bittl
Fachbereich Physik
Freie Universität Berlin
14195 Berlin, Arnimallee 14, Germany

[d] Prof. M. Taupitz
Charité – Universitätsmedizin Berlin, corporate member of Freie Universität Berlin and Humboldt-Universität zu Berlin,
Department of Radiology,
Charitéplatz 1, 10117 Berlin, Germany

Abstract

This supporting information for the article “Glycan-Induced Transchelation of Gadolinium from Magnetic Resonance Imaging Contrast Agent-Complexes” contains the EPR data from which the in the manuscript described values were derived, additionally MS data for a range of GAG-contrast agent complexes is provided.

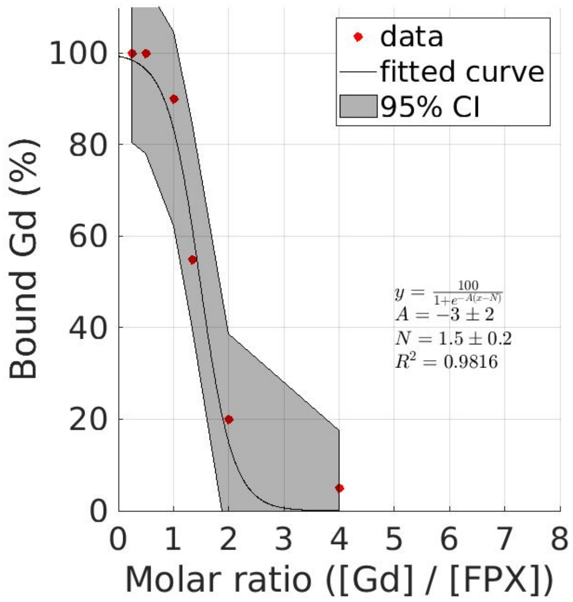

Figure S1. W-band (94 GHz) room temperature cwEPR measurements on 1 mM Gd binding to FPX in water. The fraction of the bound Gd ions is plotted as a function of molar ratio between FPX and Gd on the right. The fit with logistic function gives the stoichiometry N=[Gd]/[FPX].

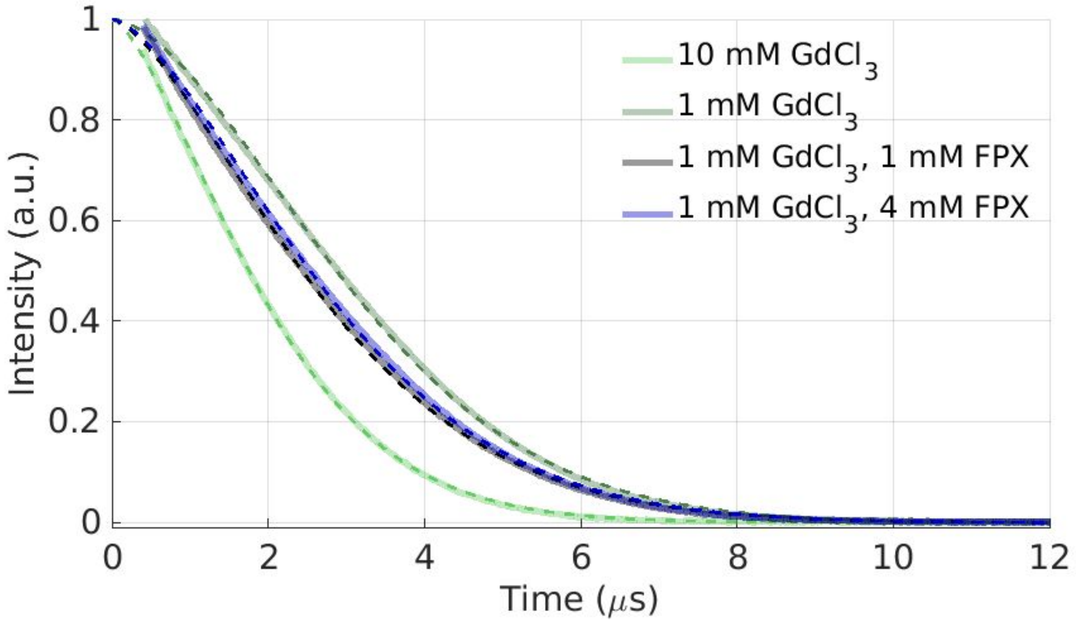

Figure S2. X-band, 10 K, spin echo decays measurement at maximum of the field swept echo (FSE) spectrum with a $\pi/2$-τ-π sequence, with a length of the π-pulse of 112 ns and τ = 200 ns. Solid lines correspond to the 10 mM $GdCl_3$ and samples with 0, 1 and 4 mM FPX together with 1 mM $GdCl_3$ in water/glycerol solution. Dashed lines represent fits with stretched exponentials.

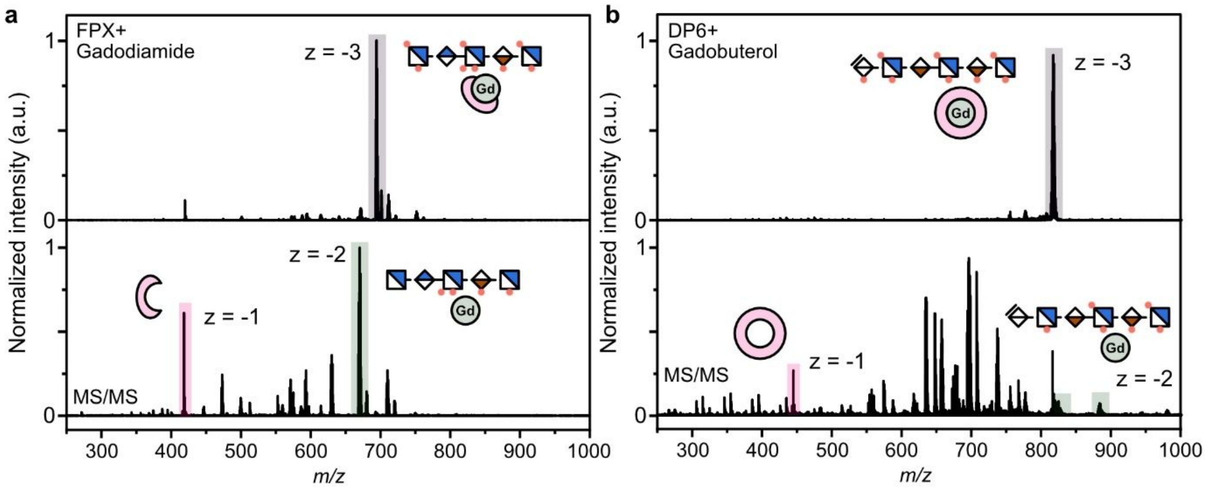

Figure S3. CID of GAG-contrast agent complexes. a) Dissociation of the FPX-Gadodiamide complex leading to the release of the gadolinium from its ligand. Due to higher needed CID energies for complex dissociation, loss of sulfate occurs prior to dissociation at unspecified positions. b) Dissociation of the GAG-Gadobutrol complex leading to the release of the gadolinium from its macrocyclic ligand but to a much lesser extent.
